# Novel prostate cancer immunotherapy with a DNA-encoded anti-prostate-specific membrane antigen monoclonal antibody

**DOI:** 10.1007/s00262-017-2042-7

**Published:** 2017-08-17

**Authors:** Kar Muthumani, Liron Marnin, Sagar B. Kudchodkar, Alfredo Perales-Puchalt, Hyeree Choi, Sangya Agarwal, Veronica L. Scott, Emma L. Reuschel, Faraz I. Zaidi, Elizabeth K. Duperret, Megan C. Wise, Kimberly A. Kraynyak, Kenneth. E. Ugen, Niranjan Y. Sardesai, J. Joseph Kim, David B. Weiner

**Affiliations:** 1 Vaccine and Immunotherapy Center, The Wistar Institute, 3601 Spruce Street, Philadelphia, PA 19104 USA; 2 Department of Pathology and Laboratory Medicine, Perlman School of Medicine, University of Pennsylvania, Philadelphia, PA 19104 USA; 3 Department of Molecular Medicine, University of South Florida Morsani College of Medicine, Tampa, FL 33612 USA; 4 Inovio Pharmaceuticals, Plymouth Meeting, PA 19462 USA

**Keywords:** Prostate cancer, Prostate-specific membrane antigen, DNA-encoded monoclonal antibodies, Immunotherapy, In vivo electroporation

## Abstract

Prostate-specific membrane antigen (PSMA) is expressed at high levels on malignant prostate cells and is likely an important therapeutic target for the treatment of prostate carcinoma. Current immunotherapy approaches to target PSMA include peptide, cell, vector or DNA-based vaccines as well as passive administration of PSMA-specific monoclonal antibodies (mAb). Conventional mAb immunotherapy has numerous logistical and practical limitations, including high production costs and a requirement for frequent dosing due to short mAb serum half-life. In this report, we describe a novel strategy of antibody-based immunotherapy against prostate carcinoma that utilizes synthetic DNA plasmids that encode a therapeutic human mAb that target PSMA. Electroporation-enhanced intramuscular injection of the DNA-encoded mAb (DMAb) plasmid into mice led to the production of functional and durable levels of the anti-PSMA antibody. The anti-PSMA produced in vivo controlled tumor growth and prolonged survival in a mouse model. This is likely mediated by antibody-dependent cellular cytotoxicity (ADCC) effect with the aid of NK cells. Further study of  this novel approach for treatment of human prostate disease and other malignant conditions is warranted.

## Introduction

Prostate cancer is the second most frequently diagnosed cancer and the sixth most deadly cancer in males worldwide [1–3]. In the USA, prostate cancer is the most commonly diagnosed cancer in males over the age of 50 years and ranks as the second deadliest cancer in males [4, 5]. Traditional treatments for prostate cancer include prostectomy, radiation therapy, chemotherapy and hormone deprivation therapy [5]. These treatments can impair the quality of life for patients and thus new approaches to combating prostate cancer are warranted [4]. Several groups are exploring methods for harnessing the immune system to recognize and kill prostate cancer cells [2]. One such effort has led to Sipuleucel-T, a licensed, autologous cellular immunotherapy for the treatment of asymptomatic or minimally symptomatic metastatic castrate-resistant prostate cancer [6]. Additional immunotherapies for prostate cancer now under development include a number of vaccine candidates, as well as approaches using targeted monoclonal antibodies (mAbs) [7].

Prostate-specific membrane antigen (PSMA) is expressed many fold higher on prostate cells than cells of other tissues, and it is considered an important clinical biomarker of prostate cancer [8–10]. Levels of PSMA are further elevated on prostate cancer cells, and studies indicate a strong correlation between increased PSMA expression and prostate cancer progression [4, 5]. PSMA expression levels can also be elevated on other malignant cells including those of urologic origin (i.e., kidney and bladder) suggesting this glycoprotein may play a role in their oncogenic progression as well [11]. In other solid tumors including colon, ovarian, breast, and kidney cancers, elevated PSMA expression has been observed on tumor neovasculature, but not normal vasculature suggesting a role for PSMA in angiogenesis [12]. Unlike prostate-specific antigen (PSA), PMSA is a membrane protein which makes it an attractive target to develop mAbs against it for diagnostic and therapeutic purposes [13]. Several therapeutic anti-PSMA mAbs have been developed, and many of these have been used in radioimmunotherapy for targeting cytotoxic radionucleotides, specifically to PSMA-expressing cells [5]. Some anti-PSMA mAbs, such as clone 2C9, have been demonstrated to mediate a therapeutic effect by promoting an antibody-dependent cellular cytotoxicity (ADCC) effect that kills prostate cancer cells [5, 14].

DNA plasmids have been used for over 25 years as a non-viral method of in vivo gene delivery, and they have been studied extensively as a platform for vaccines and gene therapy. Recently, our group has explored developing synthetic DNA plasmids as a means of delivering the genes of MAbs that neutralize infectious agents. We have reported that constructs expressing DNA-encoded monoclonal antibody (DMAb) can direct in vivo production of functional levels of antibody targeting human immunodeficiency, dengue, and chikungunya viruses in mice [15–17]. Such an approach possesses several advantages over both conventional protein-based mAbs and viral vector-based delivery of antibody genes including; (1) lower production costs; (2) the ability to generate durable, high levels of in vivo antibody production without gene integration; and (3) the ability for repeated administrations due to the non-immunogenic nature of DNA plasmids. While early applications of DNA plasmid technology suffered due to poor in vivo transgene production, recent enhancements in the design of DNA vectors along with new delivery methods including adaptive in vivo electroporation (EP) have combined to boost transgene expression to potent levels in clinical vaccine studies, without compromising safety [18].

This study describes the first application of enhanced synthetic DNA plasmid technology to deliver DNA directing the in vivo production of a human MAb for cancer immunotherapy. We designed a novel construct encoding a therapeutic anti-PSMA MAb, and we show that this plasmid expresses DMAb in vitro and in vivo in mice after EP-enhanced intramuscular delivery. The in vivo generated antibodies retain their ability to bind specifically to PSMA, and they possess ADCC activity. Finally, we show that this anti-PSMA-DMAb can control the growth of a PSMA-positive tumor in a mouse model, likely through engagement of NK cells.

## Materials and methods

### Cell lines and reagents

Cell lines used in this study were purchased from American Type Culture Collection (ATCC). The 293T(ATCC^®^CRL-3216™) and transgenic adenocarcinoma mouse prostate (TRAMP)-C2 (ATCC^®^CRL-2731™) cells were maintained in Dulbecco’s modified Eagle’s medium (DMEM) (Gibco-Life Technologies) supplemented with 10% FBS and 1% penicillin/streptomycin. Lymph node carcinoma of the prostate (LNCaP) clone FGC (ATCC^®^CRL-1740™) cells were maintained in RPMI-1640 (Gibco-Life Technologies) supplemented with 10% fetal bovine serum (FBS) and 1% penicillin/streptomycin [19]. The commercial anti-PSMA control mAb was obtained from R&D systems.

### PSMA-DMAb plasmid construction and expression confirmation

To construct the PSMA-DMAb , the genes of both the variable heavy (*V*_H_) and variable light (*V*_L_) fragments of a human anti-PSMA mAb were examined, optimized, and constructed through the use of synthetic oligonucleotides with several modifications to improve expression as previously described [15]. DNA was formulated in water for subsequent administration into mice. An empty pVax1 expression vector was used as a negative control. Cells (293T) were transfected with the PSMA-DMAb plasmid and confirmation of PSMA-DMAb binding to recombinant human PSMA was carried out by Western blot analysis. Briefly, recombinant PSMA protein (R&D systems) was run on an SDS-PAGE gel and transferred to Immobilon-PVDF membrane (EMD Millipore). Membranes were blocked for 1 h in blocking buffer (Li-Cor Biosciences) and then incubated for 1 h with either commercial anti-PSMA mAb (R&D systems), pooled day 14 sera from PSMA-DMAb plasmid-injected mice, or supernatants from PSMA-DMAb plasmid-transfected 293T cells. Membranes were washed and then incubated for 1 h with a goat anti-human IgG 680RD antibody (Li-Cor Biosciences) and washed. Protein bands were visualized by scanning membranes with a Li-Cor Odyssey CLx scanner [19].

### Mice, plasmid administration, and IgG quantification

Animal experiments were conducted in accordance with the University of Pennsylvania Animal Care and Use Committee guidelines. B6.Cg-*Foxn1* ^*nu*^/J (C57BL/6 nude) and C57BL/6 (both from Jackson Laboratory) mice were administered 100 µg of PSMA-DMAb or pVax1 plasmid in a single 50 µl intramuscular injection into the quadriceps, followed by in vivo electroporation [15]. For quantifying human immunoglobulin G1 (IgG1) levels, ELISA plates were coated with 1 µg/well of goat anti-human IgG-Fc fragment antibody (Bethyl) overnight at 4 °C. The following day, plates were washed with phosphate-buffered saline with 0.1% Tween-20 (PBS-T), blocked with 10% FBS in PBS-T for 2 h at room temperature, washed, incubated for 1 h at room temperature with the respective samples that were diluted with 1% FBS in PBS-T, washed, and incubated for 1 h at room temperature with HRP-conjugated goat anti-human kappa light chain antibody (Bethyl). SIGMAFAST OPD (Sigma-Aldrich) solution was added to wells and plates kept in dark for at least 10 min for color to develop. The enzymatic reaction was stopped with 1 N H_2_SO_4_ and plates were read at 450 nm. A standard curve was generated using purified human IgG/Kappa (Bethyl) [15]. Binding ELISA to evaluate antibody affinity followed a similar procedure except plates were coated overnight with recombinant human PSMA and a HRP-conjugated goat anti-human IgG (H + L) (Bethyl) was used as a secondary antibody.

### Flow cytometry analysis

To detect cell surface PSMA, tubes of 1.0 × 10^6^ LNCaP or TRAMP-C2 cells were washed with phosphate-buffered saline (PBS), stained with live/dead fixable violet dead cell stain (Life Technologies) for 15 min, and then washed twice with FACS buffer (PBS + 1% FBS). Cells were next incubated for 30 min at room temperature with a 1:4 dilution of day 14 sera from PSMA-DMAb plasmid-injected mice and then washed. Finally, cells were incubated in the dark for 30 min with a 1:100 dilution of PE-conjugated anti-human Fc IgG (Biolegend), followed by a final wash with FACS buffer. Samples were resuspended in 1× stabilizing fixative (BD) and analyzed the following day on an LSR18 flow cytometer (BD Biosciences). FACS analysis was performed on a gated low forward scatter and side scatter with Annexin-V FITC and PI (Thermo Fisher) following kit protocol for the effects of PSMA-DMAb sera on LNCaP cell death.

### Indirect immunofluorescence and immunohistochemistry assay

Formalin-fixed paraffin-embedded (FFPE) human tumor tissue sections (UMass Cancer Center Tissue and Tumor Bank, Massachusetts, MA) were deparaffinized with xylene and rehydrated. Antigen retrieval was performed using a 1× working solution of citrate buffer, pH 6.0 (Sigma-Aldrich), at 100 °C for 15 min. Tissue sections were blocked with 1× PBS containing 5% normal goat serum (Cell Signaling Technology) and 0.3% Triton X-100 in a humid chamber. Tissues were washed in 1× PBS and incubated with pooled day 14 PSMA-DMAb plasmid-administered mice sera diluted 1:100 in antibody diluent. Tissues were washed in 1× PBS and incubated with a 1:500 dilution of Alexa Fluor 488-conjugated goat anti-human IgG (H + L) secondary antibody (Thermo Fisher Scientific) in antibody diluent for 1 h. Cell nuclei were counterstained with Hoechst reagent (Sigma-Aldrich). Images were acquired using the Leica TCS SP8 confocal laser scanning microscope at the cell and developmental biology microscopy core, University of Pennsylvania, PA, USA. Paraffin-embedded mouse prostate tissue was subjected to antigen retrieval and deparaffinized. Slides were then fixed with acetone and washed with PBS and sections blocked using normal goat serum followed by staining with human PSMA antibody, followed by a biotinylated goat anti-mouse and completion of immunohistochemical procedure according to the manufacturer’s instructions (Vector Labs).

### Antibody-dependent cell-mediated cytotoxicity assay

ADCC activity of PSMA-DMAb was examined using Promega’s ADCC Reporter Bioassay Kit. Briefly, target LNCaP cells were incubated for 6 h at 37 °C with the engineered Jurkat effector cells and pooled day 14 sera from PSMA-DMAb plasmid-injected mice. Luciferase activity was measured by luminescence to determine ADCC activity as recommended by the manufacturer. All sera samples were tested in triplicate.

### Tumor challenge

For tumor implantation, C57BL/6 male mice were injected subcutaneously with 1 × 10^6^ TRAMP-C2 cells in the right hind flank. The experimental mice were divided into treatment groups (*n*=10). Animals were monitored for tumor growth. As tumors became detectable, electronic calipers were used to measure the length and width of the tumor and the tumor volumes were calculated by applying the following equation: ${\text {volume\;{(V)}}} \; = {\frac{4}{3}\times{3.14159}\times\left(\frac {{\text{length}}}{2}\times\frac {{\text{width}}}{2}\times\frac {{\text{width}}}{2}\right)}$. Under the University of Pennsylvania Animal Care and Use Committee guidelines mice are sacrificed when tumor diameter reaches 2 cm, or when tumors became ulcerated. Survival differences between groups were analyzed by Students *t* test, *p* > 0.05 is considered significant.

### In vivo NK cell depletion

Mice were treated for NK cell depletion on day −1 (before tumor challenge) and at days +2 and +4 after tumor inoculation with intravenous injection of 100 μl (25 μg) of either control IgG or anti-Asialo GM1 IgG (Wako Chemicals, Richmond, VA, USA) diluted in PBS. Cells were stained with anti-NK1.1 and anti-CD3 monoclonal antibodies and analyzed by flow cytometry to verify the depletion of the CD3^−^/NK1.1+ (NK) cell population in the anti-Asialo GM1-treated animals.

### Statistical analysis

GraphPad Prism 6 (GraphPad Software, Inc.) program was used for statistical analysis of the data. The data from ELISA assays are expressed as mean ± SD and are representative of at least three different experiments. Comparisons between individual data points were made using Student’s *t* test. *p* values < 0.05 were considered to be statistically significant.

## Results

### Construction and in vitro characterization of the PSMA-DMAb plasmid

**Fig. 1:**
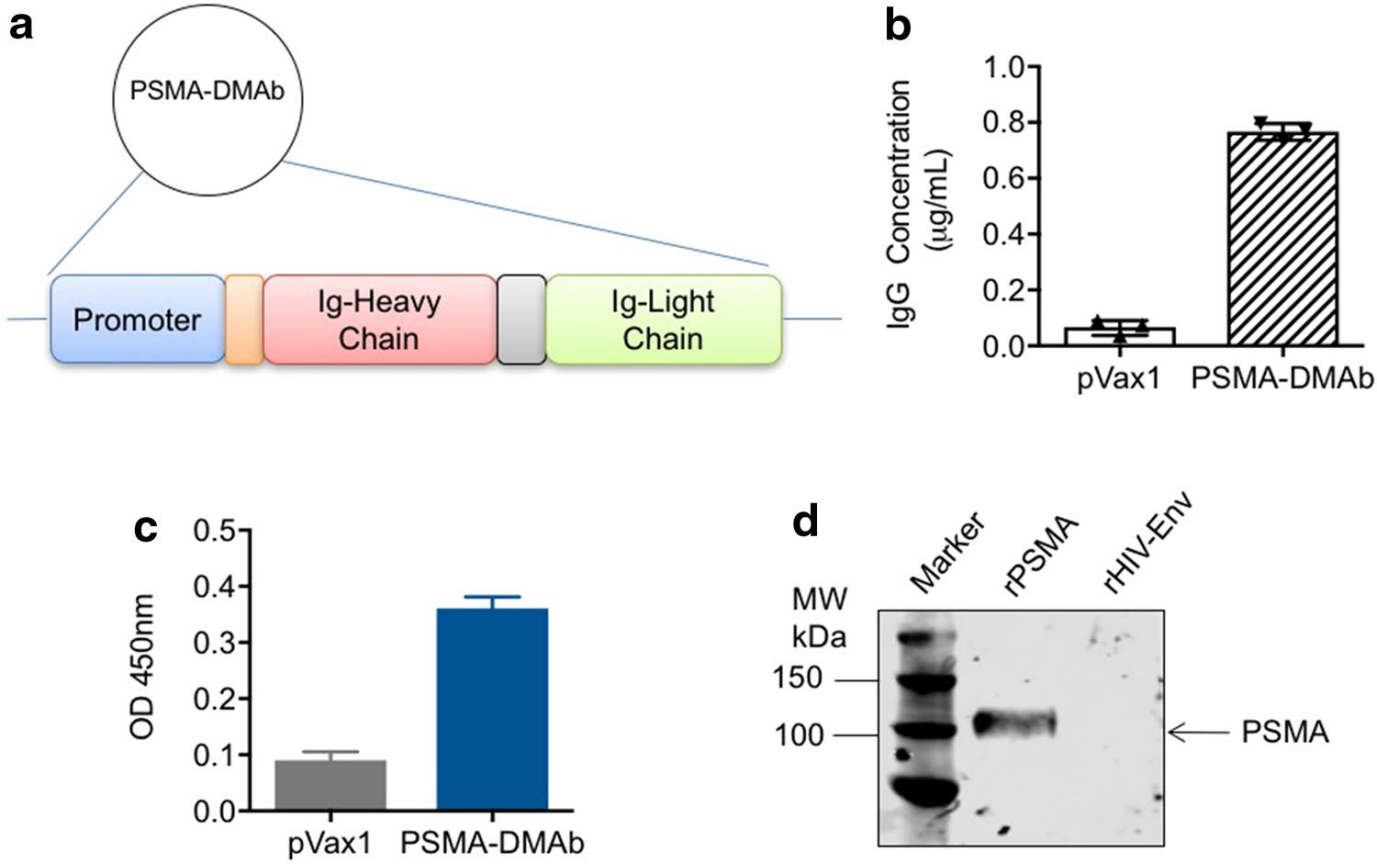
Construct design for human PSMA-DMAb and in vitro expression. **a** The schematic of the PSMA-DMAb plasmid construction. **b**–**d** Confirmation of in vitro expression and specificity of PSMA-DMAb produced antibodies in transfected 293T cells; **b** ELISA to quantitate human IgG levels in supernatants collected at 48 h post-transfection. **c** Binding specificity of supernatant IgG (1:50 dilution) to recombinant human PSMA (rPSMA) by ELISA. **d** Western blot analysis of rPSMA or recombinant HIV envelope protein (rHIV-Env) blotted with tissue culture supernatants from transfected 293T cells. *Error bars* in **b** and **c** are SDs

Human PSMA is a type II integral membrane glycoprotein that is highly expressed in prostate secretory-acinar epithelium as well as in several extra-prostatic tissues, and it possesses 86% identity and 91% similarity to mouse PSMA [20]. A plasmid capable of directing in vivo antibody production was designed by (1) creating a cassette consisting of the full-length coding sequences for the variable heavy (*V*_H_) and light (*V*_L_) immunoglobulin (*I*g) chains from the published sequence of an anti-PSMA mAb driven off a CMV promoter; (2) optimizing the cassette sequence to improve its expression; and (3) cloning the cassette into a pVax1plasmid (Fig. 1a). Antibodies targeting PSMA produced from this optimized DNA plasmid will henceforth be referred to as PSMA-DMAb.

To confirm that the plasmid directs production of fully assembled IgG, human embryonic kidney 293T cells were transfected with either empty pVax1 or PSMA-DMAb plasmid. Supernatants collected from cells at 48 h post-transfection were assayed by ELISA to quantify total human IgG levels. A concentration of nearly 800 ng/ml of human IgG was measured in supernatants of PSMA-DMAb plasmid-transfected cells (Fig. 1b). A binding ELISA performed on the same supernatants indicated that the IgG produced from PSMA-DMAb plasmid-transfected cells bound to recombinant human PSMA with high affinity (Fig. 1c). Western blot analysis further confirmed the specificity of PSMA-DMAb plasmid-derived antibodies for binding to recombinant human PSMA protein (Fig. 1d). The results indicate that the PSMA-DMAb plasmid can direct the production of anti-PSMA-specific antibodies in vitro.

### PSMA-DMAb plasmid administration generates PSMA-specific antibodies in vivo

**Fig. 2:**
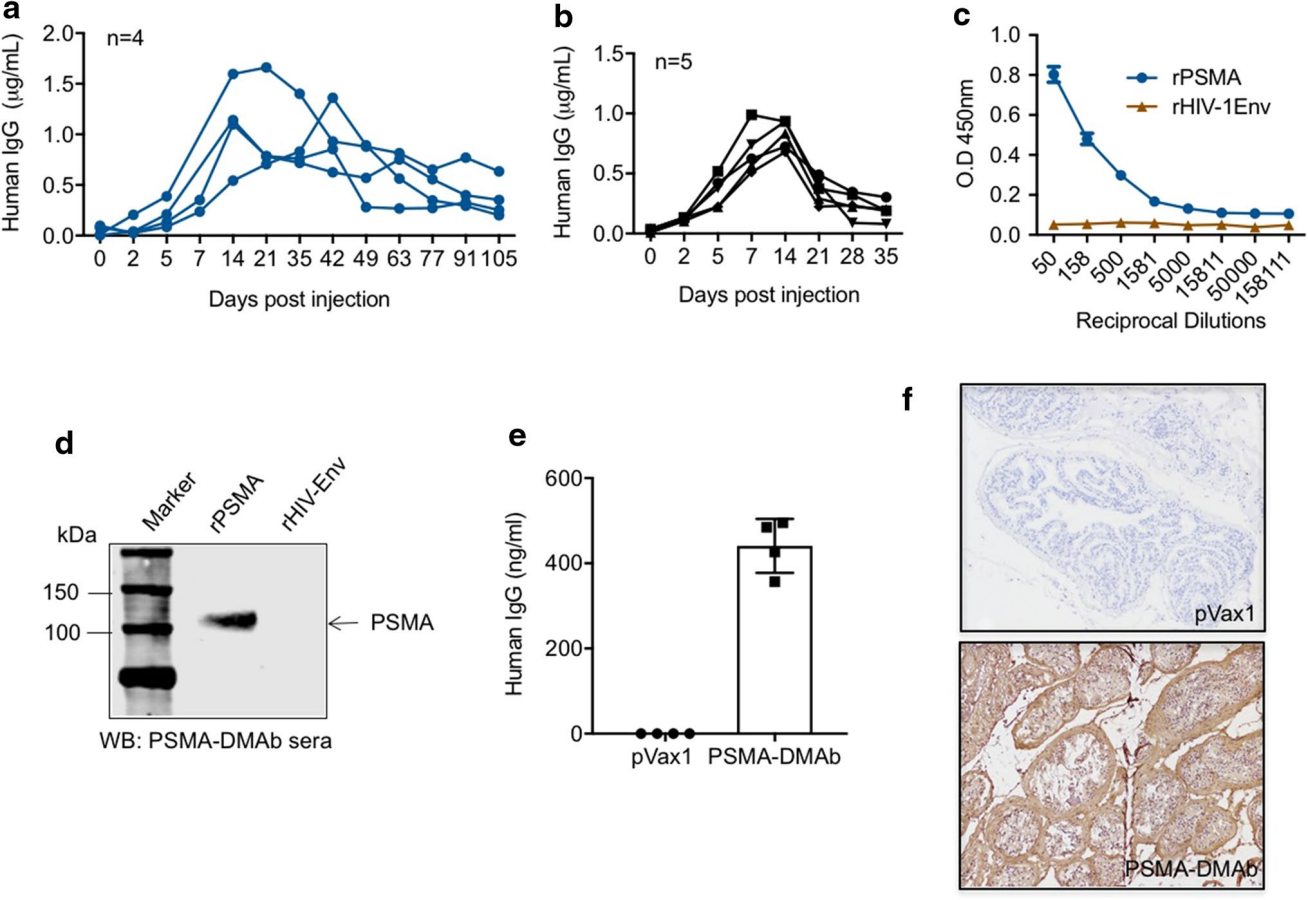
Confirmation of in vivo expression and specificity of the PSMA-DMAb in mice. Measurement of human IgG in sera from **a** immunodeficient B6.Cg-*Foxn1* ^*nu*^/J (C57BL/6 nude) mice (*n* = 5) and **b** immune-competent (C57BL/6) mice. Mice were injected with PSMA-DMAb plasmid as described in “Materials and methods” and sera levels of human IgG were measured at various time points post-injection. **c** Binding specificity, as a function of dilution, measured by ELISA in sera from PSMA-DMAb plasmid-injected nude mice collected at day 14 post-DNA administration. rPSMA and rHIV-1 Env proteins (negative control) were used as the binding antigen. **d** Binding specificity of sera from PSMA-DMAb plasmid-administered nude mice to rPSMA by Western blot analysis. rHIV-Env is used as a negative control. **e**, **f** Measurement of anti-PSMA levels in prostate tissue of PSMA-DMAb injected mice. **e** Quantification of human IgG in prostate tissue of PSMA-DMAb-plasmid and pVax1 injected mice at day 14 by ELISA. Individual IgG concentrations and mean values are shown. **f** Immunohistochemical (IHC) staining for human IgG of prostate tissues from PSMA-DMAb plasmid and pVax1 injected mice at day 14. Samples were evaluated at magnification 20×. *Scale bar* represents 100 µm

The ability of the PSMA-DMAb plasmid to direct antibody production in vivo was evaluated in both immune-deficient B6.Cg-*Foxn1* ^*nu*^/J (C57BL/6 nude) and immune-competent C57BL/6J mice. Groups of five mice received a single 100 μg injection of PSMA-DMAb plasmid intramuscularly in their quadriceps muscle followed by EP for enhanced delivery [16]. Injected mice were bled at various time points post-injection to obtain sera that was evaluated by ELISA to quantitate human IgG levels. Human IgG became detectable in sera of injected mice beginning on day 5 post-injection, with peak levels achieved at day 14 post-injection in both C57BL/6 nude (1.17 ± 0.41 μg/ml, Fig. 2a) and C57BL/6 (0.82 ± 0.11 μg/ml, Fig. 2b) mice. While elevated human IgG levels persisted in C57BL/6 nude mice beyond 50 days, the levels in C57BL/6 mice dropped to baseline values by day 35 post-injection likely due to the mouse anti-human antibody response [21, 22]. Serum collected at day 14 post-injection from PSMA-DMAb plasmid-injected C57BL/6 nude mice was evaluated by ELISA (Fig. 2c) and Western blot (Fig. 2d) to evaluate the affinity and specificity of serum IgG for recombinant human PSMA. Both assays show that the IgG in day 14 sera recognized human PSMA, but not irrelevant HIV envelope protein with high affinity and specificity, suggesting that the IgG are properly folded and functional PSMA-DMAb.

In vivo distribution of PSMA-DMAb in prostate tissue was studied in mice by harvesting tissues 7 days post-plasmid injection and performing ELISA and immunohistochemistry for IgG quantification. Prostate tissue from mice administered the PSMA-DMAb plasmid exhibited higher levels of human IgG compared to prostate tissue from empty pVax1 plasmid-injected mice as measured by ELISA of tissue homogenates (Fig. 2e). Further, prostate tissues were evaluated by immunohistochemistry staining for anti-human-Fc expression. A strong immunostaining signal was detected on the cell membranes and within the prostate for the PSMA-DMAb plasmid-injected mice, but not pVax1-treated controls (Fig. 2f). Together, these findings demonstrated that the PSMA-DMAb plasmid can direct the production of robust levels of PSMA-specific human IgG in vivo.

### In vivo generated PSMA-DMAbs bind to PSMA on prostate cancer cells

**Fig. 3:**
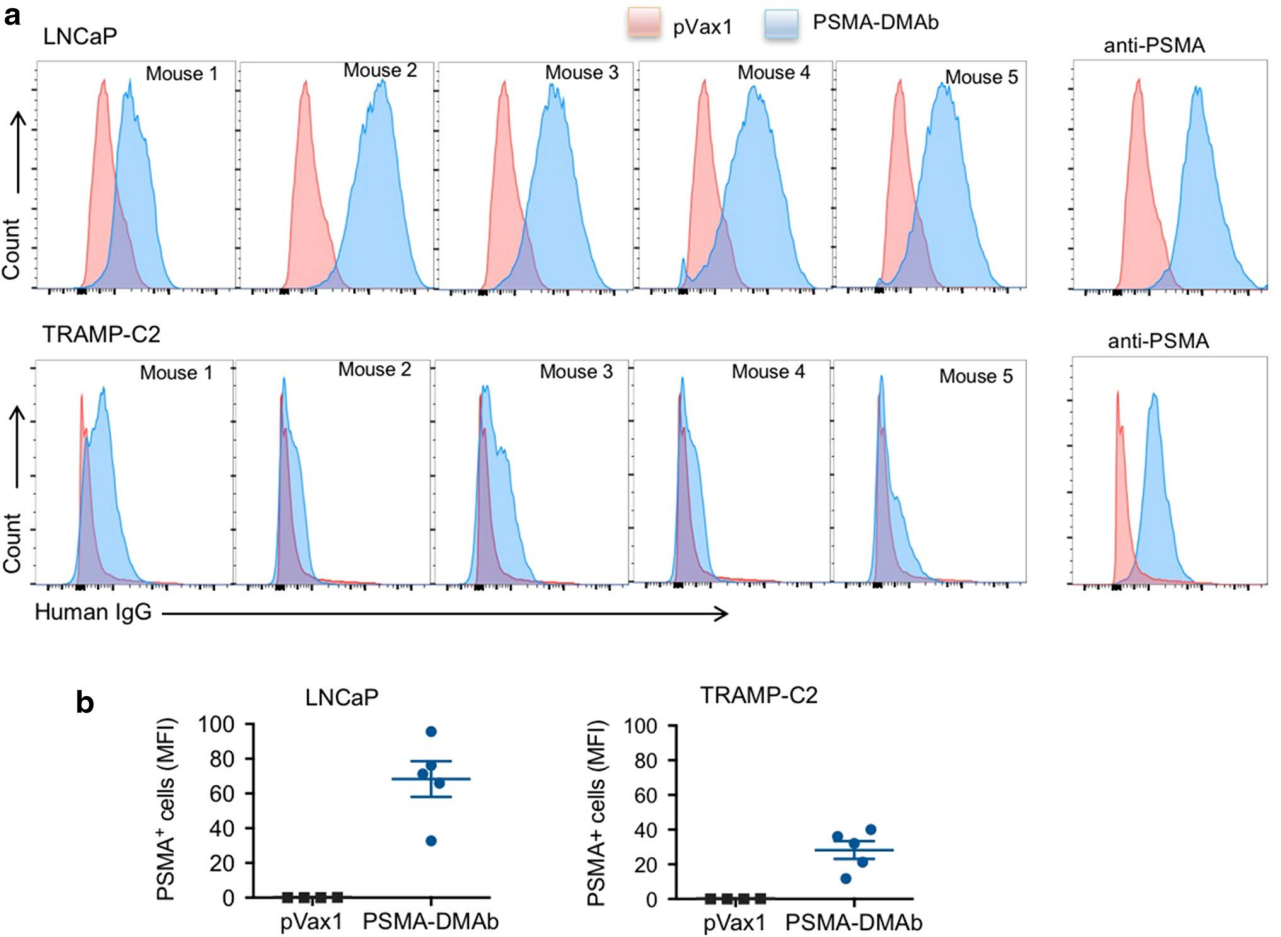
Flow cytometry analysis of PSMA-expressing LNCaP and TRAMP-C2 positive cells. **a** Overlaid histogram of PSMA expression on LNCaP (*upper panel*) and TRAMP-C2 (*lower panel*) cells. Histograms show live LNCaP or TRAMP-C2 cells stained with either day 14 sera from mice injected with pVax1 plasmid (*pink*) or day 14 sera from mice injected with PSMA-DMAb plasmid (*blue*). Representative flow cytometry of LNCaP and TRAMP-C2 cells stained with a commercial anti-PSMA antibody as a control. **b** Quantitation of MFI of PSMA binding for all mouse sera to LNCaP and TRAMP-C2 based on values measured in the five mice presented in **a**. *Error bars* indicated in **b** are SD

We next evaluated the ability of PSMA-DMAb in mouse sera to bind PSMA on tumor cells and tissues. Two PSMA-expressing prostate cancer cell lines were chosen for the initial studies: (1) LNCaP cells, derived from human prostate adenocarcinoma cells; and (2) transgenic adenocarcinoma mouse prostate (TRAMP)-C2 cells derived from a heterogeneous 32-week tumor grown in the TRAMP mouse model. Both cell lines were incubated sequentially with day 14 sera from pVax1 or PSMA-DMAb plasmid-injected C57BL/6 nude mice followed by a fluorescently labeled anti-human IgG secondary antibody. Histograms (Fig. 3a) and mean fluorescent intensity MFI (Fig. 3b) obtained from flow cytometry analysis of stained cells show that in vivo produced PSMA-DMAbs bind to both PSMA-positive tumor cell lines. No staining was observed on PSMA-negative PC3 cells (data not shown).

**Fig. 4:**
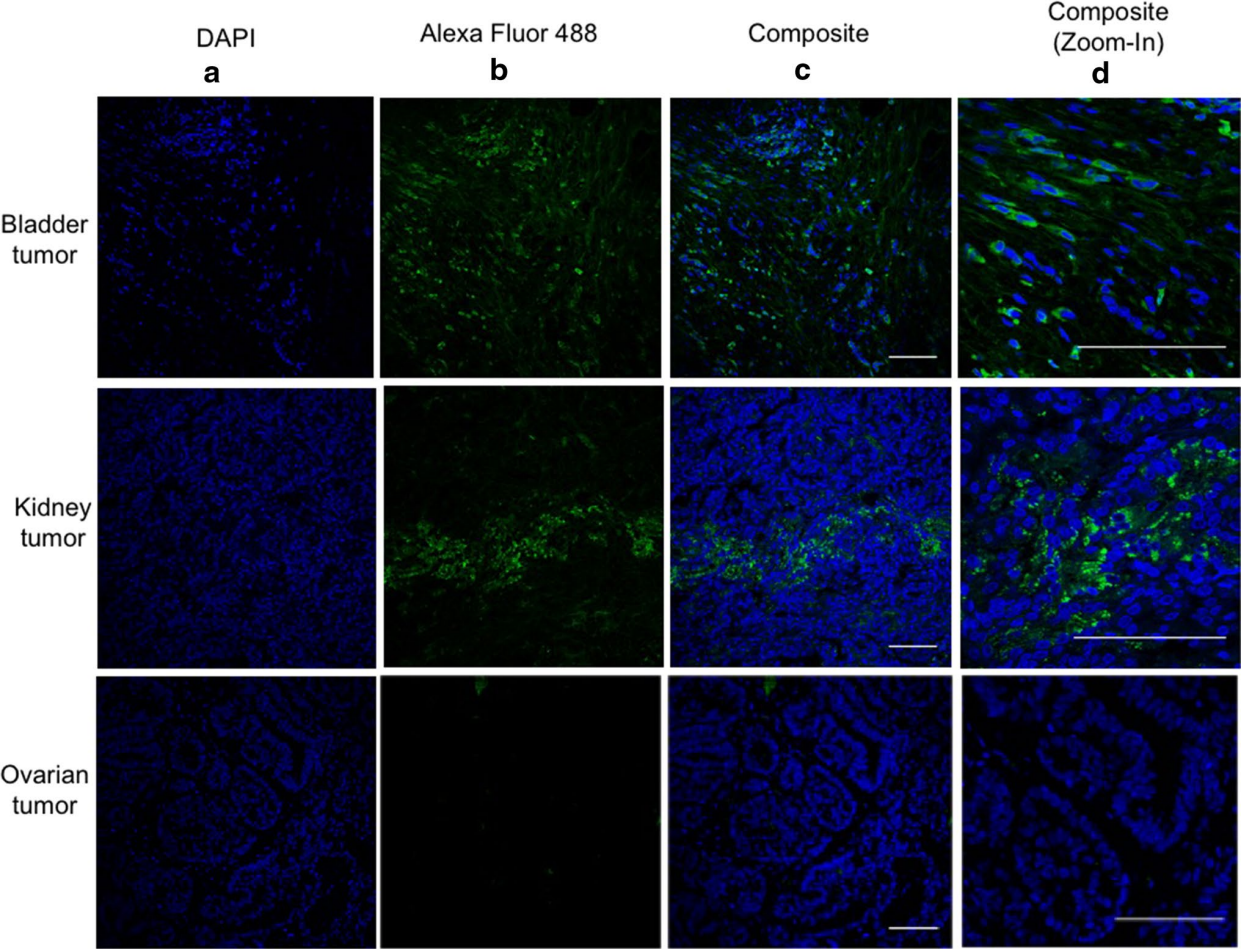
PSMA-DMAb generated antibodies bind to human bladder and kidney carcinoma tissue sections. Tissue sections were stained with pooled sera from mice collected 14 days after the administration of PSMA-DMAb plasmid. Results of the staining of the bladder and kidney carcinoma tissue sections are indicated in the *top* and *middle rows* of the *panels,* respectively, and normal ovarian tissues in the bottom. The **a** *panels* show DAPI staining of cell nuclei. The **b** *panels* show staining with anti-human IgG Alexa Fluor 488 following incubation with PSMA-DMAb sera. *Panel* **c** shows composite staining (DAPI +Alexa Fluor 488), while *panel* **d** is a magnified in photo of the composite panels

In addition to normal and cancerous prostate cells, several studies have reported PSMA expression on a wide variety of tumors, especially on tumor neovasculature [23, 24]. Immunofluorescence assays were used to evaluate the ability of PSMA-DMAb to bind to PSMA expressed on tissue sections of human bladder and kidney tumors (Fig. 4). The results show that PSMA-DMAb was able to stain cells in the bladder and kidney tumor tissue sections, but not cells in normal ovarian tissues, confirming previous reports of PSMA expression in these tumors [25]. Furthermore, the staining shows that PSMA distribution is homogeneous throughout the bladder and kidney tumor sections. This data confirms that a PSMA-DMAb retains specificity for PSMA and specifically binds PSMA on the surface of human tumor cells.

### PSMA-DMAbs possess antibody-dependent cell-mediated cytotoxicity activity

**Fig. 5:**
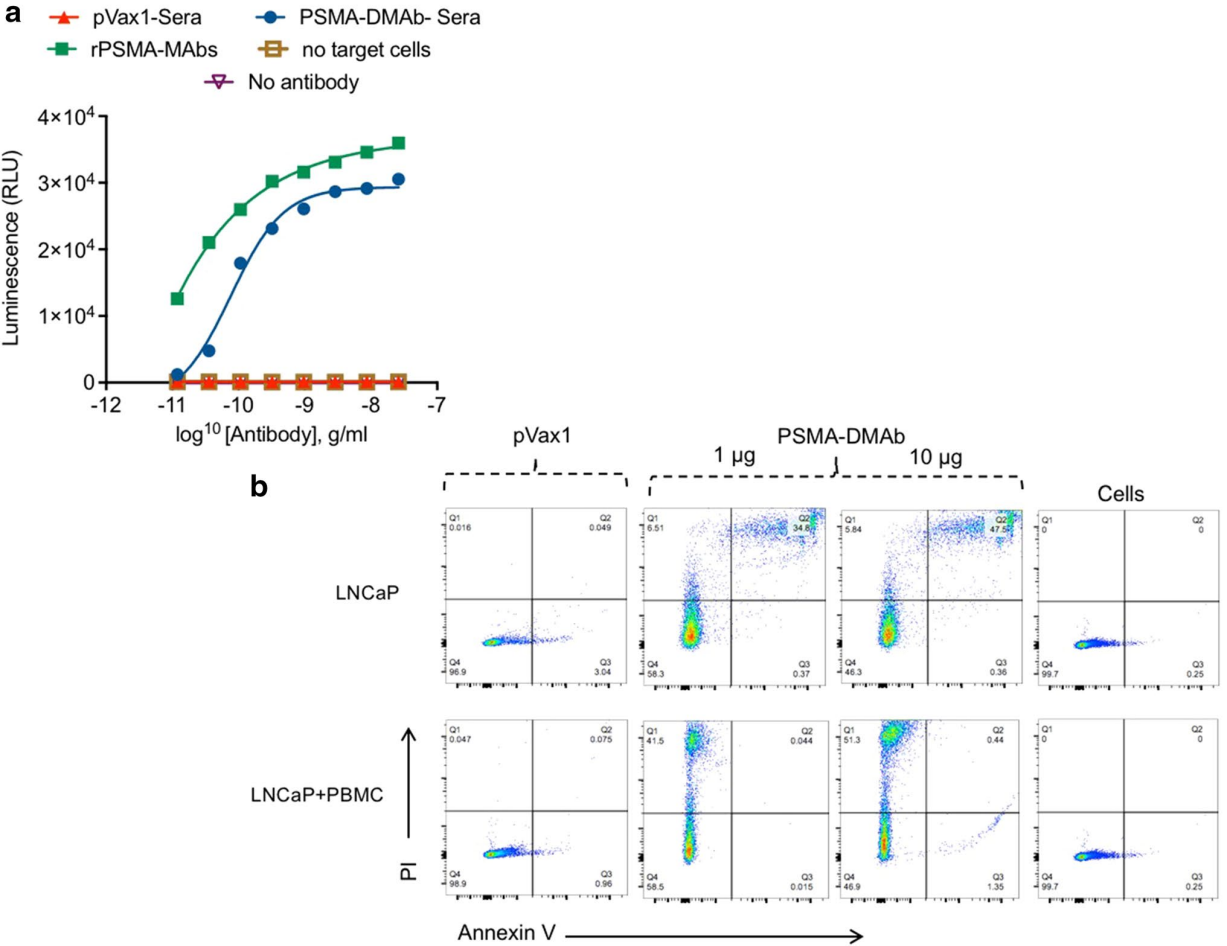
PSMA-DMAb mediates targeted death of LNCaP cells. **a** ADCC activity of PSMA-DMAb-generated antibodies. Target LNCaP cells were incubated for 6 h with the engineered Jurkat effector cells along with various dilutions of day 14 PSMA-DMAb sera samples. Negative controls such as absence of target cells (LNCaP) and no antibody, and a rPSMA-mAb as a positive control were used. Luciferase activity was measured. Results are representative data from two independent experiments. **b** Flow cytometric analysis of the effects of sera collected from PSMA-DMAb plasmid-administered mice on LNCaP cell death. Day 14 sera were incubated with LNCaP cells in the absence or presence of human PBMCs. Following washing, cells were stained with Annexin-V and propidium iodide (PI), according to the manufacturer’s assay specifications. Gated FACS scan panels are shown for the various treatments: pVax1 control (10 μg), 1 or 10 μg PSMA-DMAb and non-treated cell control. Figure illustrates a representative experiment out of two performed independently

The biological activity of PSMA-DMAb was next evaluated by using an antibody-dependent cell-mediated cytotoxicity (ADCC) mechanism of action assay [26, 27]. The assay involves incubating PSMA-expressing LNCaP cells with effector cells for 6 h in the presence of different concentrations of serum from pVax1 or PSMA-DMAb plasmid-injected mice. The effector cells are Jurkat cells that stably express high-affinity V158 FcγRIIIa and a gene for firefly luciferase driven off a nuclear factor of activated T cell (NFAT) response element [28]. The assay readout is based on activation of gene transcription in effector cells as measured by firefly luciferase production. As indicated in Fig. 5a, day 14 serum from PSMA-DMAb plasmid-injected mice mediates an ADCC effect.

As a second demonstration of the biological activity of PSMA-DMAb, flow cytometry was used to measure apoptosis and necrosis of LNCaP cells that were co-cultured with human PBMCs in the presence of sera from pVax1 or PSMA-DMAb plasmid-injected mice. The results (Fig. 5b) show that there was a statistically significant increase in apoptosis (Q3 section of the histogram) as well as necrosis (Q2 section in the histogram) for LNCaP cells co-cultured with human PBMCs in the presence of PSMA-DMAb in comparison to control pVax1 sera. Combined, these findings show that the synthetic PSMA-DMAb can bind Fc receptors and mediate an ADCC effect on tumor cells [29].

### PSMA-DMAb represses tumor growth in a TRAMP-C2 tumor challenge mouse model

**Fig. 6:**
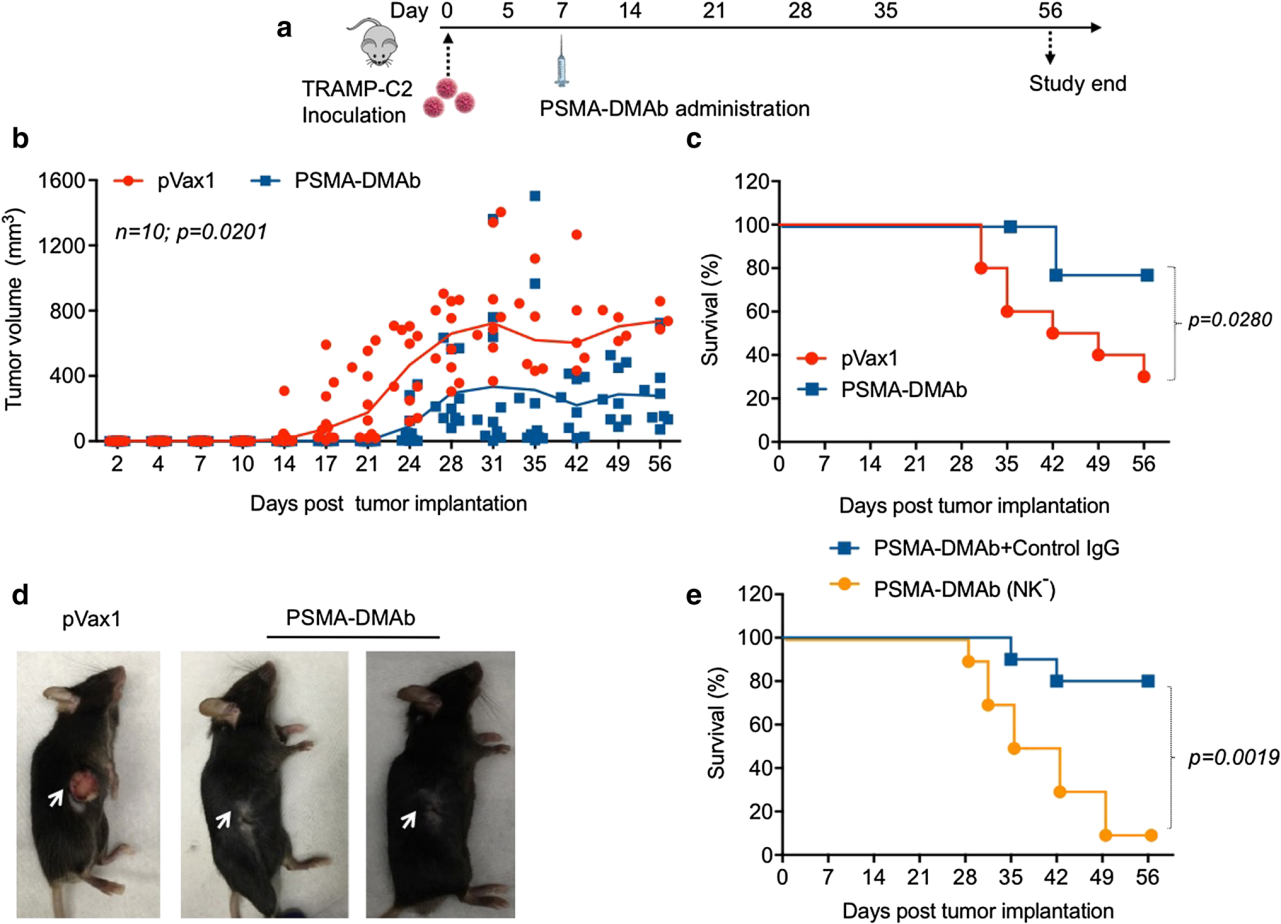
PSMA-DMAb administration induces anti-tumor immunity in a TRAMP-C2 tumor cell mouse challenge model. **a** Schema of TRAMP-C2 tumor cell administration and plasmid administration into C57BL/6 mice. Mice were administered subcutaneously 1 × 10^6^ TRAMP-C2 cells followed 1 week later by intramuscular injection of 100 μg of the DNA. **b**–**d** Assessment of tumor development in pVax1 and PSMA-DMAb plasmid-injected mice. **b** Tumor volumes (mm^3^) were measured weekly, in mice for up to 56 days post-tumor administration. **c** Kaplan–Meier curves (*n* = 10) showed the tumor survival time of mice in the pVax1 and PSMA-DMAb groups. **d** Representative mice with TRAMP-C2 tumors from pVax1 and PSMA-DMAb plasmid-injected groups at day 56 post-tumor administration. **e** Kaplan–Meier curves (*n* = 10) show the effect of NK cell depletion on PSMA-DMAb-mediated tumor survival time

In vivo functional activity of PSMA-DMAb was assessed using a TRAMP-C2 tumor challenge mouse model [30]. For this assay, C57BL/6 mice were subcutaneously implanted with 1 × 10^6^ TRAMP-C2 tumor cells and then injected 1 week later with 100 μg of either pVax1 or PSMA-DMAb plasmid by intramuscular injection with enhanced EP [30]. Mice were followed for up to 56 days with regular measurements of tumor size made on each mouse during this period (Fig. 6a). Tumors in the pVax1-treated mice began to grow at day 7–10 post-implantation, while tumors were not detectable in PSMA-DMAb-treated mice until days 15–17. Rapid tumor growth was noted for the control groups (pVax1), but the PSMA-DMAb-treated group exhibited an obvious suppression of tumor growth due to the antibody-mediated tumor-protective immunity. Over the course of the 56-day observation period, there was a statistically significant reduction in average tumor volumes (*p* = 0.0201) (Fig. 6b) and a significant improvement in survival (*p* = 0.0280) in mice receiving the PSMA-DMAb construct compared to the control mice (Fig. 6c). It is likely that this effect might be further enhanced in the absence of the mouse anti-human antibody response. Visual inspection of tumors (Fig. 6d) that developed in each group revealed that the tumors in the PSMA-DMAb group were impacted early and remained small and subdermal, while tumors in the pVax1 control group protruded out of the skin and became ulcerated.

The anti-tumor activity of many therapeutic antibodies including ADCC and antibody-dependent cellular phagocytosis (ADCP) is dependent on the interaction of the IgG-Fc domain with Fc gamma receptors (FcγRs) on effector cells. Natural killer cells express high levels of FcγRs, therefore we also examined the contribution of NK cells to the observed effects of PSMA-DMAb on tumor growth. Previous studies have reported that human IgG can bind to all activating mouse FcγRs and can induce ADCC/ADCP with mouse NK cells and mouse macrophages [29]. Groups of mice were treated with either control IgG or the NK cell-depleting anti-AGM1 IgG antibody and then implanted with TRAMP-C2 cells. One week later, mice were given a single injection of either pVax1 or PSMA-DMAb plasmid and were subsequently evaluated for tumor growth up to 56 days. There was a rapid onset of tumor development, accelerated tumor growth, and decreased survival in PSMA-DMAb-immunized, NK cell-depleted mice (*p* = 0.0019, Fig. 6e), but not in those pretreated with the control IgG. Taken together, these data demonstrate that PSMA-DMAb can exert a profound therapeutic effect on a PSMA-expressing tumor in vivo, supporting the possible application of this therapy for the treatment of prostate cancer.

## Discussion

The work presented here describes the construction and characterization of a novel DNA plasmid-based delivery system that can be used to generate protective levels of a therapeutic mAb in vivo. A DNA plasmid encoding the *V*_H_ and *V*_L_ segments of a human anti-PSMA mAb was constructed and demonstrated to direct the expression of full-length, antigen-specific IgG in vitro and in vivo following electroporation-enhanced injection into the muscles of mice. PSMA is highly expressed on prostate carcinoma as well as other tumor cells, and it is considered an attractive target for antibody-based therapy due to its expression on the surface of cells. PSMA-DMAb in the serum of mice injected with PSMA-DMAb plasmid was able to bind to PSMA on the surface of the TRAMP-C2 and LNCaP prostate tumor cell lines and to sections of bladder and kidney tumors. Serum antibody levels of 1–2 μg/ml were achieved in mice injected with the PSMA-DMAb plasmid by day 14 post-administration, and the antibody remained detectable in the sera for several weeks. Importantly, PSMA-DMAb retained the ability to recognize PSMA on the surface of implanted tumor cells and to mediate a potent anti-tumor response in vivo, due at least in part through interacting with NK cells to mediate ADCC/ADCP of tumor cells. ADCC has been hypothesized to be the major mechanism mediating the anti-tumor activity of mAbs targeting diverse malignancies [7].

Several mAbs targeting tumor-specific antigens or immunomodulatory molecules are in use or under development for cancer immunotherapy regimens, but there are impediments to their widespread use [7, 31]. One of the primary impediments involves the cost of the treatment regimen stemming from the laborious, time-consuming manufacturing and purification processes associated with making these protein-based drugs [2, 7, 14]. Additionally, multiple infusions of mAbs are often required to attain and maintain their efficacy, which imposes further cost and logistical constraints on patients [31]. Given these challenges, alternative approaches to generate and deliver mAbs are important. Gene-based administration approaches are focused on delivering the genes encoding protective antibodies so that the antibodies can be generated in vivo in a sustained manner. Several groups have developed viral vectors for delivery of mAb genes and have shown that these vectors can be used to drive production of mAbs in vivo [2, 13]. However, viral vector delivery of genes carries its own challenges, such as high development and distribution costs as well as the potential for neutralization of gene delivery and the inability to re-dose patients because of immune responses generated against the viral vector.

In this regard, the DNA plasmid-based delivery system described here possesses many unique advantageous features for use as a specific patient treatment. Primary among these is the potential for significantly lower costs stemming from lower manufacturing costs of DNA plasmids, as well as lower distribution costs because DNA is more stable and simple to produce. Synthetic DNA vectors delivered into muscle or skin with the aid of adaptive electroporation can produce high and durable levels of in vivo transgene expression without integration, and there is abundant clinical data that speaks to its favorable safety profile [18]. Since DNA plasmids are non-immunogenic, multiple administrations of the same or different plasmids can be contemplated for delivery. This feature is particularly important if serum antibody levels decrease or another antibody treatment is required.

This is the first report describing the use of a DNA plasmid-based delivery system to direct in vivo generation of a therapeutic mAb that targets a relevant oncology target, PSMA. It is also the first report to illustrate functional engagement of host NK-immune clearance by a DNA-vectored mAb. Due to the flexibility of this platform, combination of DMAb plasmids with other anti-cancer treatments or immunotherapy agents is important to consider. Furtherr study of this approach for neoplastic disease is warranted.

## Abbreviations

ADCC: Antibody-dependent cellular cytotoxicity
ADCP: Antibody-dependent cellular phagocytosis
ATCC: American Type Culture Collection
DMAb: DNA-encoded monoclonal antibodies
DMEM: Dulbecco’s modified Eagle’s medium
DAPI: (4′,6-Diamidino-2-phenylindole)
Env: Envelope
EP: Electroporation
FBS: Fetal bovine serum
Fc: Fragment crystallizable region of an antibody
FcγR: Fc gamma receptor
FDA: Food and Drug Administration
EP: Electroporation
ELISA: Enzyme-linked immunosorbent assay
FFPE: Formalin-fixed paraffin embedded
IgG: Immunoglobulin G
LNCaP: Lymph node carcinoma of the prostate
MAb: Monoclonal antibodies
MFI: Mean fluorescent intensity
NFAT: Nuclear factor of activated T cells
NK cells: Natural killer cells
PBS: Phosphate-buffered saline
PBS-T: Phosphate-buffered saline with Tween-20
PSA: Prostate-specific antigen (define in text)
PSMA: Prostate-specific membrane antigen
rHIV-env: Recombinant HIV-1 envelope
rPSMA: Recombinant human prostate-specific membrane antigen
SD: Standard deviation
TRAMP-C2: Transgenic adenocarcinoma mouse prostate
VH: Variable immunoglobulin G heavy chain
VL: Variable immunoglobulin G light chain
